# The role of Big Five personality domains and facets in musical sensibility: a twin study

**DOI:** 10.1038/s41598-025-95661-z

**Published:** 2025-04-19

**Authors:** Heidi Marie Umbach Hansen, Bruno Laeng, Olav Mandt Vassend, Nikolai Olavi Czajkowski, Tor Endestad, Anne Danielsen, Espen Røysamb

**Affiliations:** 1 Department of Psychology, University of Oslo, Forskningsveien 3A, 0373 Oslo, Norway; 2 RITMO Centre for Interdisciplinary Studies in Rhythm, Time and Motion, University of Oslo, Oslo, Norway; 3 Department of Psychology, PROMENTA Research Center, University of Oslo, Oslo, Norway; 4 Department of Mental Health and Suicide, Norwegian Institute of Public Health, Oslo, Norway; 5 Department of Neuropsychology, Helgeland Hospital, Mosjøen, Norway; 6 Department of Musicology, University of Oslo, Oslo, Norway; 7 Division of Mental and Physical Health, Norwegian Institute of Public Health, Oslo, Norway

**Keywords:** Musical sensibility, Personality, Big Five, Twin design, Psychology, Human behaviour

## Abstract

Musical sensibility can be understood as a propensity to be emotionally and aesthetically engaged by music and may constitute a key feature of a multidimensional definition of musicality. Yet, the nature of this construct is only just beginning to be understood. In a sample of adult Norwegian twins (N = 2592), we aimed to establish whether interindividual variability in musical sensibility may partially be attributable to personality, both in terms of the Big Five personality domains and their lower-order facets, as well as the role of genes and environments. Phenotypic analyses demonstrated that the personality domains of open-mindedness (and the facet aesthetic sensitivity in particular), agreeableness (and the facet compassion), and negative emotionality were all significantly associated with and predictive of musical sensibility. Multivariate biometric twin models further revealed that these relations were driven mainly by genetic influences, accounting for 50–100% of the observed covariance, whereas non-shared environmental influences accounted for the rest. Moreover, genetic correlations of musical sensibility with personality traits were substantial, and particularly strong for open-mindedness, pointing to considerable overlap in the biological mechanisms underlying the two traits. These findings situate musical sensibility within a broader network of psychological dispositions, possibly linked together via common affective systems.

## Introduction

The power of music to sound emotionally expressive and have its way with our emotions and its intrinsically rewarding value are likely to be some of the main reasons why engaging with music is considered one of life’s greatest sources of pleasure^1^. Music’s affective properties are also likely to contribute to the beneficial associations between music engagement and health and wellness characteristics^2–4^, including quality of life and well-being^5,6^. Accordingly, affective and aesthetic aspects of music have received sustained scientific attention, and there is increased focus on understanding why some people are more attuned and responsive to these factors than others. In this context, many studies have labeled, used, and interpreted different constructs and measures to investigate individual differences in the experience of musical affect. The present study is centered on a construct that we refer to as *musical sensibility*^7^, assumed to be a stable disposition representing the tendency to seek out and to be emotionally and aesthetically engaged by music—or put simply, the extent to which music makes us *feel*.

The nature of this construct is only just beginning to be understood. This is in contrast to various other musical dispositions, such as musical sophistication^8–11^ and music reward sensitivity^12,13^, where fundamental dimensions of personality such as the Big Five traits^14^ have been found to be associated with individual differences. We here suggest that interindividual variability in musical sensibility may thus also be partially attributable to personality, both in terms of broad domains and lower-order facets. However, little is known about the strength and nature of these associations, particularly the underlying genetic and environmental architecture. In the present study, we seek to reduce this knowledge gap by focusing on the role of the Big Five personality traits in the understanding of the phenomenon of musical sensibility, both empirically (e.g., by investigating genetic associations) and theoretically (by expanding and refining the construct of musical sensibility).

### Characterizing and measuring musical sensibility

Our construct of musical sensibility builds upon a recent and comprehensive self-report measure: The Module 2 of the Music Use and Background Questionnaire (MUSEBAQ), which was developed by Chin et al.^15^ in a data-driven fashion across three independent studies. This module, originally labeled ‘Music Capacity,’ was specifically intended to measure individual differences in “sensitivity or capacity to respond to music”^15^ across four subscales, namely emotional sensitivity (ten items), personal commitment (six items), memory and imagery (four items), and listening sophistication (four items). We have recently examined the underlying latent structure and the genetic and environmental basis of these four dimensions^7^. However, given their predominantly affective nature, we adopted the collective term *musical sensibility—*with ‘sensibility’ sometimes defined as “the quality of being readily and strongly affected by emotional or artistic influences and experiences; emotional awareness; susceptibility or sensitivity *to*, keen awareness *of”*^16^*.* Importantly, this is not a novel term as it has been used by other researchers with overlapping meanings, such as Callen^17^, who emphasized the close tie between music’s emotive power and aesthetic experiences in relation to musical sensibility. Using traditional factor analytical approaches and the classical twin design (CTD), we found support for a robust, highly reliable, and well-defined common factor of musical sensibility, which was also strongly heritable, with genetic effects accounting for 64% of the variance. Based on these initial findings, we proposed a general musical sensibility construct, the nature of which was interpreted as a basic musical disposition reflecting global emotional and aesthetic responsivity to music, as indexed by items such as “*Listening to music fills me with emotion* “ and “*Music can produce feelings of wonder and fascination in me.*” Of note, we will use the term ‘aesthetic’ in relation to the appreciation of beauty (e.g., in art and nature) but do not claim that this constitutes a distinct class of emotion^18^.

The concept of musical sensibility should be considered to belong to a family of related dispositions that resonate with the contemporary multidimensional view of musicality, wherein musicality is described as the ability to perceive, produce, and enjoy music^19^. This inclusive notion of musicality has been paralleled by the development of a rich repository of questionnaires aimed at assessing musical skills and behaviors in the general population (e.g.^10,12,15,20,21^). Some are quite targeted in their focus, such as the Barcelona Music Reward Questionnaire (BMRQ)^12^, which taps into strongly hedonic aspects of music experiences (music seeking, emotion evocation, mood regulation, social reward, and sensory-motor). Others are conceptually broader, such as the Goldsmiths Musical Sophistication Index (Gold-MSI)^10^, and is useful for capturing skilled musical behaviors (e.g., self-reported perceptual- and singing abilities), as well as active music engagement, music training, and emotional engagement with music. Our conception of musical sensibility also shares common ground with certain constructs used in empirical aesthetics, such as Desire for Aesthetics (defined as a global desire for or responsiveness to aesthetic stimuli^22^), but can be distinguished from others, like Musical or Visual Aesthetic Sensitivity^23,24^, which refers to individual differences in the extent to which weighing of certain stimulus attributes (e.g., complexity) explains variability in an individual’s aesthetic appreciation of the stimuli.

### Personality and musicality

Personality refers to dispositional patterns of thinking, behaving, and feeling that are generally stable across an individual’s lifespan. Within personality trait theory, the Big Five^25^ has become one of the most frequently adopted models, along with the widely used Big Five Inventory (BFI)^14,26^. The model consists of five broad personality dimensions (domains) and a set of conceptually narrower facets. In the BFI-2^27^, which was utilized in the present context, these include the domains of *extraversion* (characterized by facets such as sociability, assertiveness, and energy level), *agreeableness* (pro-social tendencies, with facets including compassion, respectfulness, and trust), *conscientiousness* (preference for order and structure, such as organization, productiveness, and responsibility), *negative emotionality* (or neuroticism, tendency to experience negative emotions, with facets like anxiety, depression, and emotional volatility), and *open-mindedness* (or openness to experience, characterized by facets such as intellectual curiosity, aesthetic sensitivity, and creative imagination).

Current research into the personality correlates of musical dispositions and engagement can broadly be categorized into three main directions. The first, and probably the most studied association, concerns personality traits and music preferences (e.g.^28–34^). These studies indicate reliable, albeit small, associations between personality traits and preferences for certain musical styles and attributes^34^. The strongest relationships have been reported for open-mindedness and various musical styles, indicating that individuals scoring high on open-mindedness tend to enjoy music more in general. Two recent studies further illuminated facet-level associations between personality and music preferences^35,36^. For instance, using the International Personality Item Pool-300^37^, Butković and Žauhar^35^ found two of the six open-mindedness facets to emerge as particularly important predictors of music preferences: the facet of artistic interest (i.e., sensitivity to and interest in art and beauty) positively predicted preferences for Reflective and Complex (e.g., jazz, classical) and Upbeat and Conventional (e.g., pop, country) musical styles, whereas the liberalism facet (i.e., the proclivity to reassess traditional social, religious and political values) negatively predicted preferences for Upbeat and Conventional and Regional (i.e., styles that were characteristic for the specific region the study was conducted in, such as patriotic styles).

The second stream of research has emphasized the role of personality traits in music involvement and skilled musical behavior (e.g., music training, practice, and musical sophistication). Open-mindedness has been reported to significantly predict music training (e.g.^38,39^), perceptual abilities^8,40,41^, and overall musical sophistication^8–11^. One of the few studies thus far assessing personality at the level of facets in this regard is a comprehensive study by Greenberg et al.^8^. They examined the relationship between the 10 facets of the original version of the BFI^14^ and musical sophistication, as measured by the Gold-MSI. Several facets were found to be significant predictors of general musical sophistication, with openness to aesthetics having the strongest unique effects (β = 0.43), while the effects of other facets were more modest (i.e., β < 0.10).

Of particular interest to the present study is the third line of research, which has focused on the link between personality and several affective aspects of musical experiences. These studies have shown that certain personality traits appear to be associated with both quantitative and qualitative aspects of emotional responses to music^42,43^. For example, Liljeström et al.^42^ found that the self-reported intensity of music-induced emotions while listening to music was significantly and positively correlated with agreeableness, extraversion, and open-mindedness (*r*s = 0.55, 0.40, and 0.34, respectively). They further showed that people rating high on negative emotionality tended to experience more negative than positive emotions when listening to music (e.g., sadness-melancholy), whereas the opposite pattern was observed for individuals scoring high on extraversion, open-mindedness, and agreeableness (e.g., more positive feelings like happiness-elation).

Open-mindedness, together with trait empathy, has also been reported to be positively associated with the enjoyment of sad music^43,44^. Further, the proclivity to experience peak emotional responses from music, commonly referred to as music “chills”, has repeatedly been linked to open-mindedness^45–48^. Another study^49^ employed the Big Five Aspects Scale^50^ to examine individual differences in aesthetic experiences in response to the arts in general. The findings indicated that individuals with high scores on the openness facet, which in this context specifically relates to aesthetics, creativity, and emotional sensitivity (rather than the intellect facet), strongly predicted both a global factor of aesthetic experiences and more specific aspects, like chills and absorption^49^. In contrast, the feeling of being ‘touched’ was most strongly linked to the compassion facet of the agreeableness domain. A few studies have investigated the link between personality and overall music reward sensitivity, as measured using the BMRQ^12,13^. In one study including all Big Five personality traits and the BMRQ, Wang et al.^13^ found positive and significant independent effects for four of the five personality domains on overall music reward, including agreeableness (β = 0.27), open-mindedness (β = 0.20), negative emotionality (β = 0.14), and conscientiousness (β = 0.14). In contrast, extraversion was non-significant (β = 0.03).

In sum, the current research shows a relation between personality and various musical dispositions and experiences. Most studies point to a particularly central role of open-mindedness, specifically concerning music involvement, an association that has been explained in terms of greater interest and sensitivity for art and aesthetic experiences by individuals scoring high on open-mindedness. Yet, more nuanced patterns of associations emerge concerning the affective aspects of music. Regarding the latter, personality traits that lean more towards intra- and interpersonal emotional tendencies (e.g., negative emotionality and agreeableness), tend to become more relevant.

However, most research suffers from important limitations, such as the way personality has been assessed (e.g., only selected personality domains or short-form measures). Crucially, because personality, in this context, has rarely been investigated at the facet level, there is limited knowledge about which facets drive the associations between broader personality domains and the various musicality traits. Moreover, most studies have relied on relatively small samples consisting of young adults. Given the existing evidence of age-related changes in music engagement, as well as the associations with personality^51^, there is a need to expand the age group in which this relationship is studied.

### Genetics of musicality and personality

The scientific study of the biological and genetic basis of musicality has flourished over the past decades, showing that genetic factors appear to play an important role in most traits relating to musicality (average heritability of 42%^52^), including musical sensibility (heritability of 64%)^7^. Likewise, decades of research have shown that personality is genetically influenced, typically accounting for approximately 40–50% of variance^53–55^. Findings from the broader behavioral genetic literature on musicality also indicate a genetic overlap between certain musicality traits and other non-musical psychological characteristics. For instance, there is evidence suggesting broad pleiotropic genetic effects, i.e., genetic influences that affect two or more traits, between intelligence and traits such as music practice, auditory discrimination, as well as perceptual- and motor timing abilities^56–58^.

Yet only one previous twin study^59^ has examined the relationship between Big Five-related personality traits and a musicality phenotype. In this study, intrinsic motivation, IQ, open-mindedness, and self-reported music flow proneness, measured by a sub-scale of the Swedish Flow Proneness Questionnaire^60^, were included as predictors of music practice, and of these, only open-mindedness and music flow were found to be significant (βs = 0.19 and 0.41, respectively). A multivariate twin analysis further revealed that genetic factors, relative to non-shared environmental influences, had stronger influences on the associations between open-mindedness, music flow, and music practice, accounting for 61–76% of the phenotypic correlations. While this study provided important initial insight into the origins of the association between specific aspects of personality and music practice, fundamental questions remain unanswered, particularly regarding other personality traits and the relationship to the affective and aesthetic aspects of musicality, or what we defined here as musical sensibility. Thus, there is a need to expand previous findings by including a broader set of personality traits and a comprehensive measure of musical sensibility, using psychometrically well-validated instruments.

### The present study

The main objective of the present study is to provide a foundational and more in-depth understanding of the nexus of personality and musical sensibility, both phenotypically and etiologically. Specifically, we aimed to (a) establish which personality domains and facets have unique and substantively meaningful contributions to musical sensibility, and thereby gain insights into dispositions that characterize general musical sensibility; (b) estimate the magnitude of the effects of genes and environments on the covariation of musical sensibility with personality traits, and to quantify the degree of overlap in the underlying genetic and environmental factors. To this end, we used correlation and regression analyses to assess the phenotypic relationship among the personality domains, their corresponding facets, and a global musical sensibility score. Next, multivariate biometric twin modeling was used to decompose the variation in each trait and the covariation between them into genetic and environmental influences and to estimate genetic and environmental correlations.

## Results

### Descriptive statistics, correlations, and regression analyses

An overview of descriptive statistics for each variable, as well as bivariate correlations and results from the multiple regression analyses of the Big Five personality domains and facets, are displayed in Table 1. The relationship between personality and musical sensibility was first assessed at the domain level. All personality domains except conscientiousness were significantly correlated with musical sensibility (Table 1, block I), with open-mindedness exhibiting the strongest relationship (*r* = 0.50, *p* < 0.001). In the multiple regression, in which the five domains were tested simultaneously, three of the five domains had significant unique effects after controlling for age and sex (agreeableness, negative emotionality, and open-mindedness; Table 1, block I), with open-mindedness having the strongest unique effects (β = 0.48, *p* < 0.001). This full model accounted for 29% of the variance in musical sensibility.

**Table 1:** Descriptive statistics, correlations, and regression values for the personality dimensions, facets, and musical sensibility scores.

Personality domains	Descriptives			Associations			
	Range	Mean	sd	Corr	p value	β	p value
Block I							
Age						− 0.05	0.007
Sex						− 0.003	0.872
Extraversion	1–5	3.39	0.68	**0.11**	< 0.001	0.03	0.138
Agreeableness	1–5	4.00	0.50	**0.11**	< 0.001	**0.12**	< 0.001
Conscientiousness	1–5	4.06	0.61	− 0.04	0.072	− 0.03	0.124
Negative emotionality	1–5	2.50	0.81	**0.17**	< 0.001	**0.21**	< 0.001
Open-mindedness	1–5	3.45	0.70	**0.50**	< 0.001	**0.48**	< 0.001
Adjusted *R*^*2*^						0.29	< 0.001
Musical sensibility	1–5	3.15	0.77				
Block II							
Age						− 0.07	< 0.001
Sex						− 0.04	0.045
Extraversion							
E1 sociability	1–5	3.34	0.94	0.08	< 0.001	0.03	0.224
E2 assertiveness	1–5	3.16	0.81	0.05	0.044	− 0.001	0.985
E3 energy level	1–5	3.68	0.78	**0.15**	< 0.001	0.06	0.027
Agreeableness							
A1 compassion	1–5	4.31	0.62	**0.19**	< 0.001	**0.11**	< 0.001
A2 respectfulness	1–5	4.05	0.59	0.02	0.316	0.02	0.326
A3 trust	1–5	3.65	0.74	0.05	0.033	0.01	0.744
Conscientiousness							
C1 organization	1–5	3.99	0.84	− 0.05	0.045	0.03	0.239
C2 productiveness	1–5	4.00	0.75	− 0.02	0.340	0.002	0.933
C3 responsibility	1–5	4.20	0.60	− 0.03	0.128	− 0.08	0.002
Negative emotionality							
N1 anxiety	1–5	2.70	1.00	**0.16**	< 0.001	0.06	0.024
N2 depression	1–5	2.21	0.90	**0.11**	< 0.001	0.07	0.015
N3 emotional volatility	1–5	2.60	0.94	**0.15**	< 0.001	0.05	0.040
Open-mindedness							
O1 intellectual curiosity	1–5	3.51	0.77	**0.35**	< 0.001	0.07	0.003
O2 aesthetic sensitivity	1–5	3.20	1.04	**0.53**	< 0.001	**0.44**	< 0.001
O3 Creative imagination	1–5	3.63	0.79	**0.29**	< 0.001	0.05	0.02
Adjusted *R*^*2*^						0.33	< 0.001

Descriptive statistics, correlations, and regression models were based on a sample consisting of single twins and one individual from each full twin pair. Correlations and standardized regression coefficients higher than ± 0.10 significant at *p* < 0.01 in bold. Sex was coded as: men = 0, women = 1.

*corr* correlations; *sd* standard deviation, *β* standardized regression coefficient.

Next, we performed the same set of analyses for the personality facets (Table 1, block II). Twelve of the 15 facets were significantly correlated with musical sensibility, with correlations ranging from − 0.05, *p* = 0.045 (C1 organization) to 0.53, *p* < 0.001 (O2 aesthetic sensitivity). Results from the multiple regression showed that nine facets had significant effects, including one facet from the extraversion domain (E3 energy level), one from the agreeableness domain (A1 compassion), one from the conscientiousness domain (C3 responsibility), and all three facets from both the negative-emotionality- and open-mindedness domains. Of these nine, however, only two facets had substantial betas above 0.10 (A1 compassion and O2 aesthetic sensitivity). Combined, 33% of the variance in the musical sensibility score was explained by this model.

Of note, we also performed a sensitivity analysis, wherein the analyses were repeated using open-mindedness- and aesthetic sensitivity scores in which the BFI-2 item that explicitly mentioned music (i.e., item 20: *Is fascinated by art, music, or literature*) was excluded. Results showed that the associations with musical sensibility remained significant and substantial, both at the domain- (*r* = 0*.*46, *p* < 0.001; β = 0.44, *p* < 0.001) and facet level (*r* = 0*.*46, *p* < 0.001; β = 0.34, *p* < 0.001).

### Multivariate biometric models

To focus on personality traits with substantive effects, we chose to include only the personality domains and facets that had regression betas > 0.10 and with p < 0.01 in the biometric twin models. Sex- and age-residualized scores were used in all twin analyses since these influences can inflate twin-pair correlations^61^. No significant differences were found in the variable means nor variances within twin pairs or across zygosity groups (all *p’*s ≥ 0.15; Supplementary Table S1).

We calculated intraclass correlations across zygosity groups for each variable (Table 2). The monozygotic (MZ) intraclass correlations were always higher than the dizygotic (DZ) intraclass correlations, indicating additive genetic (A) influences. Moreover, the pattern of intraclass correlations generally indicated potential dominance genetic (D) effects (i.e., 2x*r*DZ < *r*MZ), except for the agreeableness variable. In this case, potential shared-environmental (C) influences were indicated (i.e., 2x*r*DZ > *r*MZ). The unique environmental (E) variance component is always included since it also contains measurement errors.

**Table 2:** Twin-cotwin correlations for musical sensibility, personality domains, and selected facets.

	MZ	DZ
Musical sensibility	0.64	0.28
Personality domains		
Extraversion	0.57	0.16
Agreeableness	0.46	0.27
Conscientiousness	0.46	0.27
Negative emotionality	0.48	0.22
Open-mindedness	0.64	0.27
Personality facets		
A1 compassion	0.36	0.14
O2 aesthetic sensitivity	0.59	0.25

*MZ* monozygotic, *DZ* dizygotic.

Next, a four-variate Cholesky model was fitted to estimate the unique and shared genetic- and environmental influences on the broad personality domains and musical sensibility. This model encompassed the personality domains of open-mindedness, negative emotionality, and agreeableness, in addition to the musical sensibility score. The first three variables were the personality traits (entered from the strongest to the weakest regression coefficient). The musical sensibility score was entered last in the model to allow calculation of the proportion of genetic and environmental variance in musical sensibility that is unique versus that which is shared with personality.

**Fig. 1:**
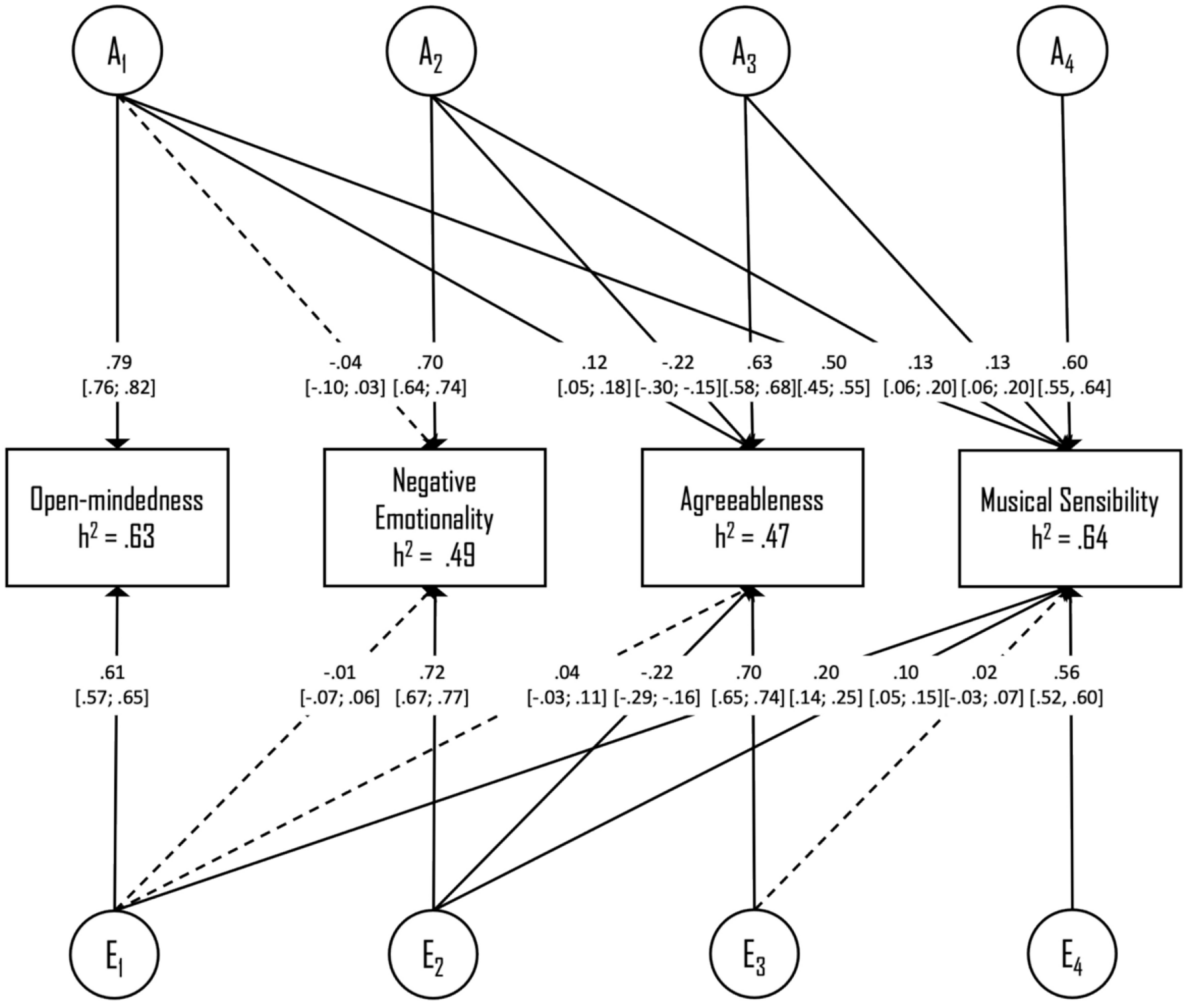
Path diagram of the best-fitting AE Cholesky model for the personality domains and musical sensibility. Path coefficients—and their 95% confidence intervals—are presented as standardized parameter estimates. Non-significant parameters are highlighted with dotted arrows. *A* Additive genetic factors; *E* Non-shared environmental factors.

Goodness-of-fit measures are summarized in Table 3, block I. Model fitting results showed that both the shared-environmental and the dominance genetic variance components could be dropped without a significant loss of fit (AE model), whereas the additive genetic variance component could not (E model). Thus, the best-fitting model according to the Akaike Information Criterion (AIC) was the AE model. In this model, heritability and the 95% confidence intervals were estimated to be 0.63 [0.57; 0.67] for open-mindedness, 0.49 [0.41; 0.55] for negative emotionality, 0.47 [0.40; 0.53] for agreeableness, and 0.64 [0.59; 0.68] for the musical sensibility score. Standardized path coefficients for the best-fitting four-variate AE model of the personality domains and musical sensibility are illustrated in Fig. 1. As depicted, the genetic paths from negative emotionality and agreeableness to musical sensibility were both significant after accounting for the effects of open-mindedness, whereas the non-shared environmental paths were only significant for open-mindedness and negative emotionality.

**Table 3:** Model fitting results for tests of the full ACE/ADE and the nested models. Best-fitting AE models are highlighted in boldface type.

Model	EP	Δ − 2LL	Δ df	Δ AIC	*p*
Block I: Personality domains					
ACE	34				
ADE	34	− 2.20	0	− 2.20	
** AE**	**23**	**1.01**	**11**	**− 20.99**	**1.000**
E	12	639.97	22	595.97	< 0.001
Block II: Personality facets					
ACE	21				
ADE	21	− 1.38	0	− 1.38	
** AE**	**14**	**1.02**	**7**	**− 12.98**	**0.995**
E	7	428.90	14	400.89	< 0.001

AE model with genetic (A) and non-shared environmental influences (E).

*AIC* Akaike Information Criterion; *df* degrees of freedom; *EP* estimated parameters; *p* probability; − *2LL* negative two times the log-likelihood.

As a sensitivity analysis, we also fitted a Cholesky model comprising all five personality domains (entered from the strongest to the weakest regression coefficient). As illustrated in Supplementary Table S2, neither the genetic nor environmental paths from extraversion and conscientiousness were significant after accounting for the effects of open-mindedness, negative emotionality, and agreeableness.

**Fig. 2:**
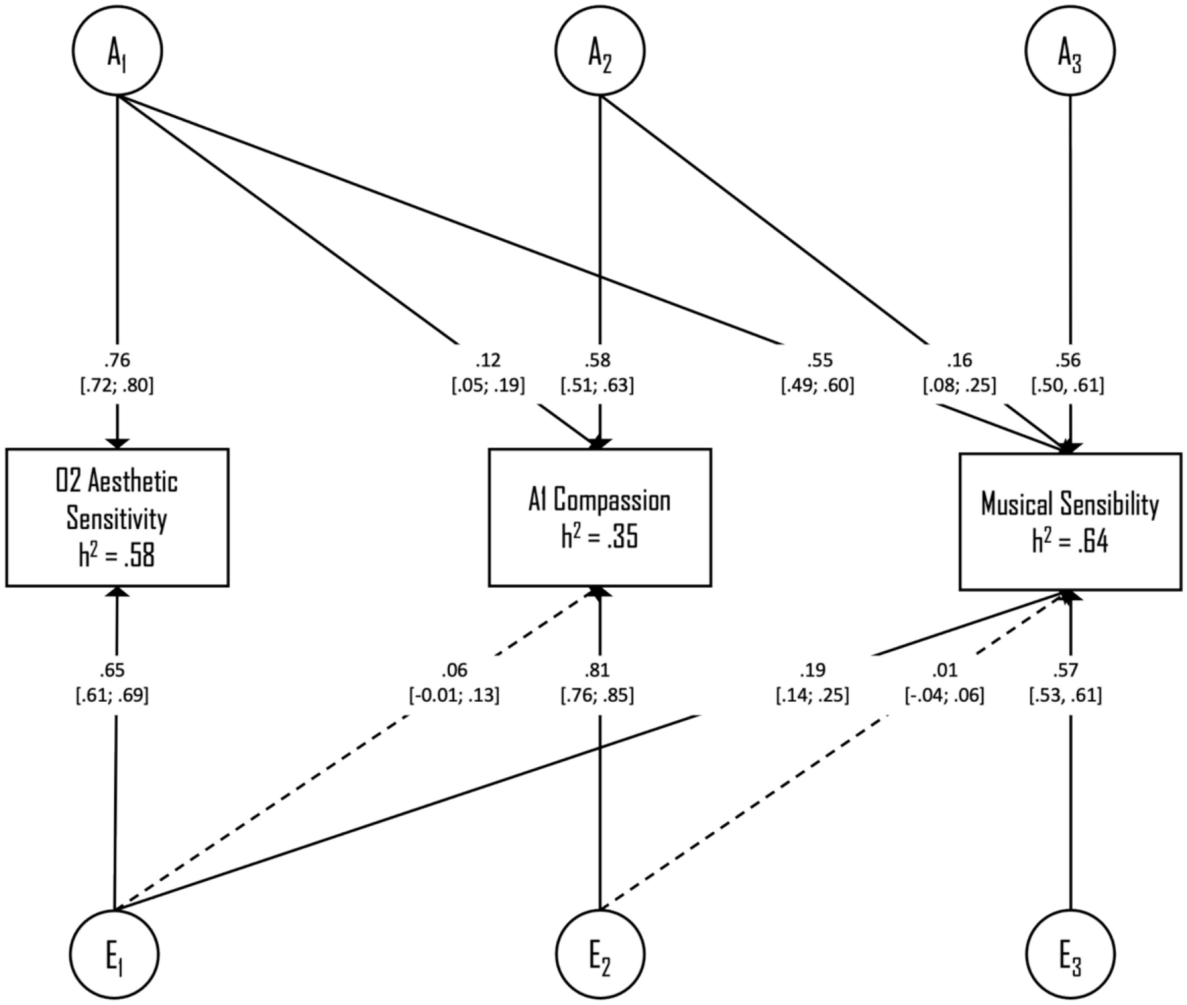
Path diagram of the best-fitting AE Cholesky model for the personality facets and musical sensibility. Path coefficients—and their 95% confidence intervals—are presented as standardized parameter estimates. Non-significant parameters are highlighted with dotted arrows. *A* Additive genetic factors; *E* Non-shared environmental factors.

Next, based on the regression analysis of the personality facets, we tested a three-variate Cholesky model encompassing 02-aesthetic sensitivity, A1-compassion, and musical sensibility. Results from the model comparisons (Table 3, block II) showed that the best-fitting model according to the AIC was the AE model, suggesting once more that the most parsimonious account of the data was a model containing only additive genetic- and unique environmental effects. The estimated heritability and the 95% confidence intervals were 0.58 [0.52; 0.63] for the O2-aesthetic sensitivity facet, 0.35 [0.27; 0.42] for the A1-compassion facet, and 0.64 [0.59; 0.68] for the musical sensibility score. Standardized path coefficients for the best-fitting three-variate AE model of the personality facets and musical sensibility are depicted in Fig. 2. Of note, the genetic path from A1-compassion to musical sensibility remained significant after accounting for the effects of O2-aesthetic sensitivity.

To obtain a better understanding of the underlying genetic and environmental etiology of the musical sensibility and personality links, bivariate heritability estimates, as well as pairwise genetic- and environmental correlations, were calculated based on the best-fitting AE models. Whereas bivariate heritability estimates reflect the amount of the observed (phenotypic) covariance that is due to genetic factors, pairwise genetic and environmental correlations index the extent to which the underlying genetic and environmental factors on one trait also influence the other^62^.

**Fig. 3:**
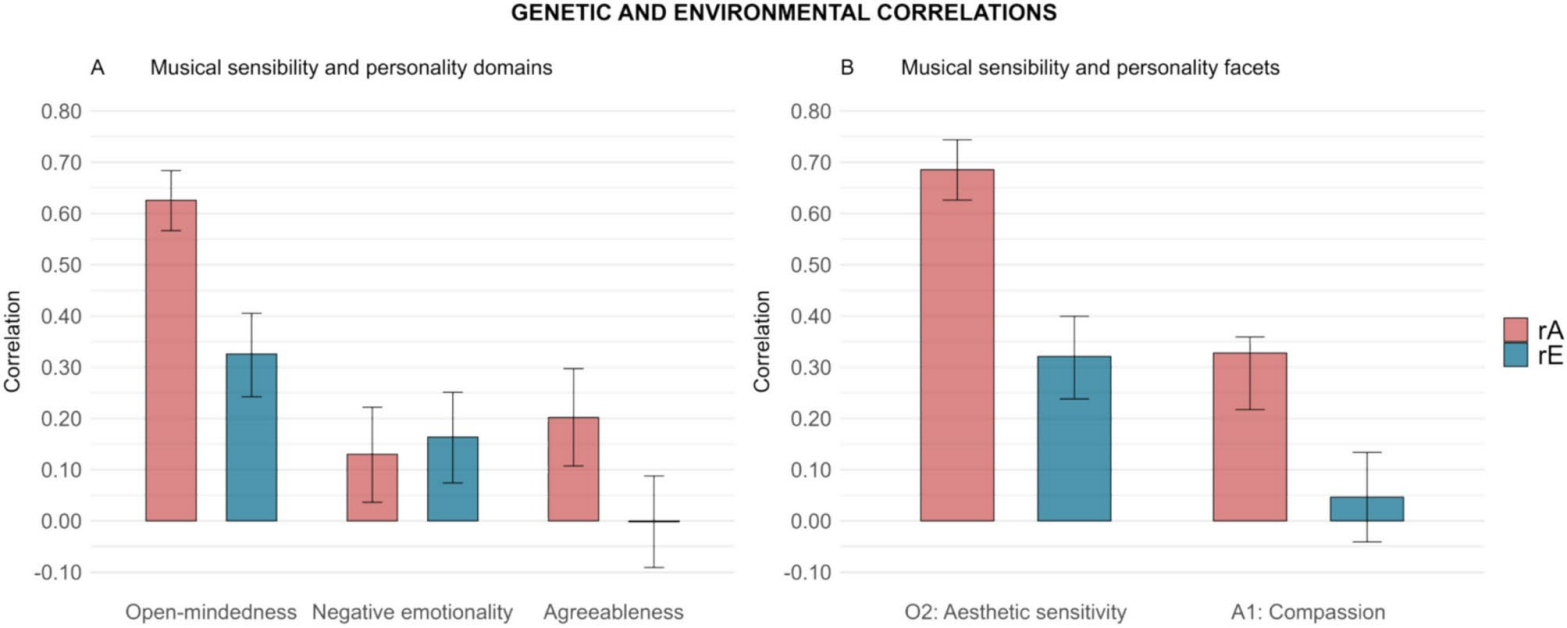
Pairwise genetic- and environmental correlations for general musical sensibility and personality domains (**A**) and facets (**B**). *rA* additive genetic correlations; *rE* non-shared environmental correlations. Variables are sorted based on the order they were entered in the biometric twin models.

Bivariate heritability estimates between musical sensibility and agreeableness (and the compassion facet) were close to 100% (both at the domain and facet level), negative emotionality about 50%, and the open-mindedness domain and the aesthetic sensitivity facet were about 77% and 76%, respectively. For the two latter personality traits, the rest was explained by non-shared environmental influences. Figure 3 depicts the pairwise genetic- and environmental correlations and the 95% confidence intervals between musical sensibility and the personality domains (Fig. 3A) and facets (Fig. 3B). The genetic correlations of musical sensibility were all significant and particularly strong for the open-mindedness domain (0.63) and facet (0.69), small to moderate for the agreeableness domain (0.20) and facet (0.33), and small for the negative emotionality domain (0.13). In addition, significant environmental correlations were found between musical sensibility and the open-mindedness domain and facet (0.33 and 0.32, respectively), as well as musical sensibility and negative emotionality (0.16), but were non-significant for agreeableness at both the domain and facet level (− 0.002 and 0.05, respectively).

**Fig. 4:**
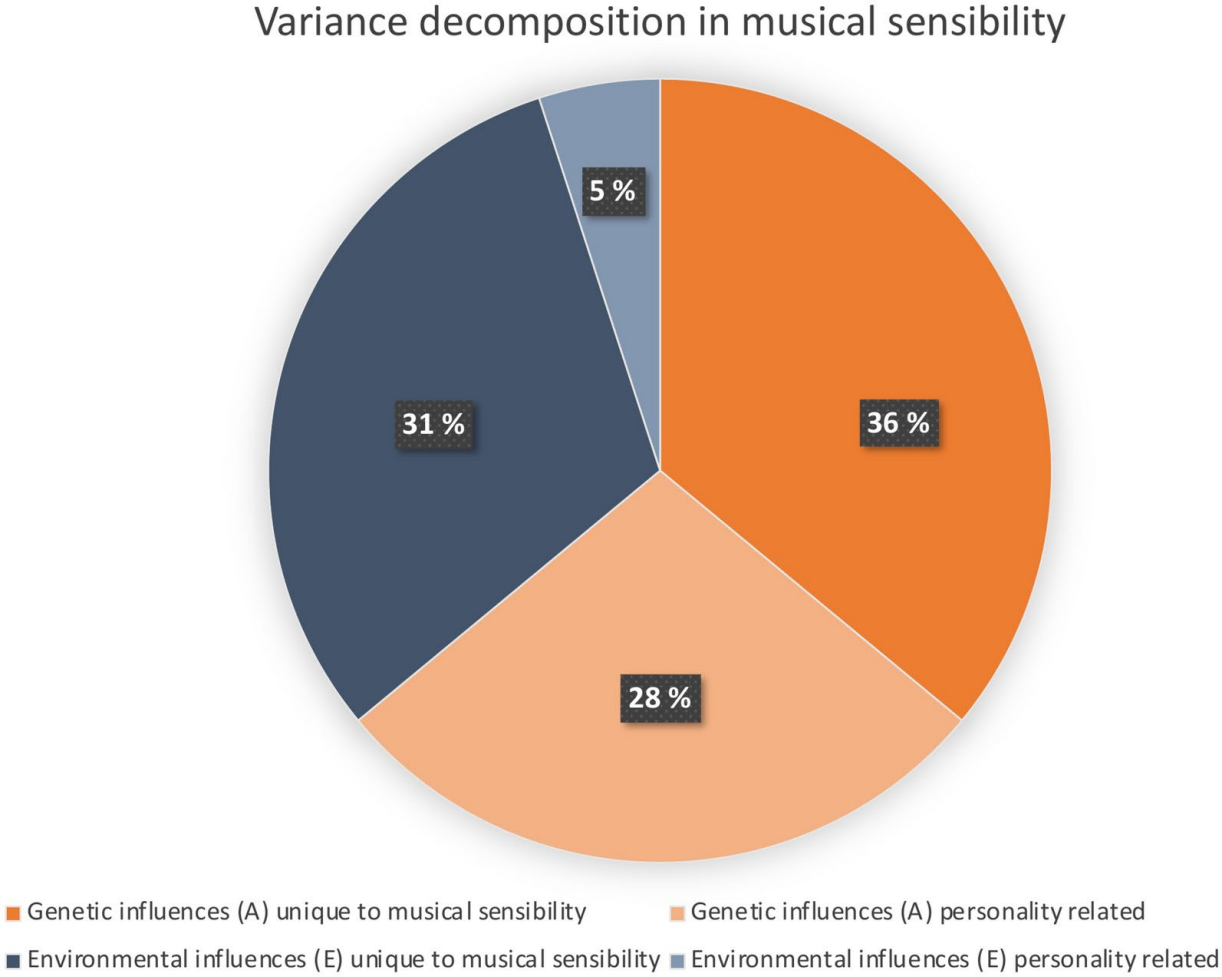
Genetic and environmental variance decomposition in musical sensibility.

Finally, we decomposed the genetic and environmental variance components of musical sensibility into personality-related influences (agreeableness, negative emotionality, and open-mindedness), versus what can be considered to be unique to musical sensibility (Fig. 4). A total of 28% of the variance in musical sensibility could be attributed to personality-related genetic factors, whereas 36% was explained by genetic factors that were unrelated to personality. This means that of the total heritability of musical sensibility (h^2^ = 0.64), about 44% was due to personality-related genetic factors, and 56% was due to genetic factors independent of personality. Additionally, there were also personality-related environmental influences, accounting for 5% of the variance, whereas 31% was unique to musical sensibility. Thus, out of the total environmental variance in musical sensibility (e^2^ = 0.36), 14% were related to personality and 86% were specific to musical sensibility, including random measurement error.

## Discussion

The present article presents a genetically informed study examining in detail the association between the Big Five personality domains and facets and individual differences in musical sensibility. Using data from a large Norwegian twin sample, we found open-mindedness to be most strongly associated with musical sensibility, followed by negative emotionality, and then agreeableness. At the facet level, only aesthetic sensitivity from the open-mindedness domain and compassion from the agreeableness domain had substantively meaningful unique effects, whereas facets from the negative emotionality domain had limited relevance (i.e., βs < 0.10).

The most novel and important result of the present study, however, regards the etiological factors that influence the association between personality and musical sensibility. Overall, the amount of shared genetic- and environmental variance with personality-related factors was 28% and 5%, respectively. As such, our results point to a key role of overlapping genetic factors in these associations. Given the emotional and aesthetic nature of these traits, a reasonable interpretation of these findings is that musical sensibility is related to personality through common emotional and aesthetic processes.

### Phenotypic associations between personality and musical sensibility

The strong association between open-mindedness and musical sensibility was expected, given the extant empirical support for this link. Further, of the three open-mindedness facets, only the aesthetic sensitivity facet had significant and substantial effects (β = 0.44, *p* < 0.001), which is consistent with previous results related to general musical sophistication^8^. In this previous study, however, open-mindedness was measured with two facets (openness to aesthetics and openness to ideas), compared to the conceptually broader measure used here (covering three facets). Thus, the present results share similarities with but also extend prior findings, corroborating a key role of the aesthetic sensitivity facet.

The positive relationship between agreeableness and musical sensibility corresponds nicely with the results reported by Wang et al*.*^13^, where agreeableness was identified as a strong trait predictor of music reward sensitivity, and the results by Silvia et al.^49^ who found that compassion strongly predicted the feeling of being ‘touched’ by art. To our knowledge, however, research into the link between a similar musicality construct, and the compassion facet per se, is still lacking. Representing the affective component of agreeableness, the compassion facet can be characterized by active emotional concerns for the well-being of others^27^. Highly compassionate people are therefore more attuned to social cues and the emotional states of others. Given that music inherently relates to emotional expressions and social-emotional behaviors (e.g., dancing, romantics), it seems consistent that compassionate individuals may also be more responsive to the emotions conveyed or evoked by music. Indeed, as described earlier, agreeableness has been associated with strong and positive emotional responses to music^42^ and to experience more intense emotional responses to music in general^63^.

The inclusion of interpersonal emotionality as a feature of musical sensibility also accords with literature linking musical affect to social-emotional processes, e.g.^64,65^. Indeed, preliminary neuroimaging results^66^ found higher white matter connectivity between sensory processing areas (superior temporal gyrus) and the insula and medial prefrontal cortex (areas that have often been implicated with socio-emotional processing) in individuals who are prone to experience intense emotional responses to music (i.e., chills), as compared to controls matched for confounding variables such as IQ and open-mindedness. Interestingly, research on the related construct of trait empathy and its facet empathic concern has shown that, in addition to promoting the enjoyment of sad music^43^, empathic concern can be positively associated with social-relational feelings like being moved or touched by music^67^. Moreover, another study reported strong, positive associations between empathy and music reward in both children and adults^68^.

Our finding that negative emotionality positively predicts musical sensibility can be interpreted in light of work highlighting the strong emotional bond between the two^69^. However, in contrast to personality traits like agreeableness and open-mindedness, which are often associated with positive music-induced emotions and aesthetic experiences (e.g., eudemonic feelings of connectedness, beauty, crying out of awe), individuals high on negative emotionality appear to be particularly susceptible to experiencing negatively valenced emotions in music (e.g., sadness)^63,70^ and intense emotional responses to music, including crying (or feeling like crying out of sadness)^71^. Negative emotionality has even been found to be positively associated with aversive musical experiences, i.e., strong negative emotional- and physical responses to disliked music, whereas an inverse relationship was found for open-mindedness, agreeableness, and extraversion^72^. Yet, evidence suggests that individuals high on negative emotionality nevertheless use music specifically for its emotional properties (e.g.,^73^) and display a preference for so-called sad music^74^.

The observed weak associations between extraversion and musical sensibility are consistent with past research on music reward sensitivity^13^ and with some studies on general musical sophistication^8,9^, whereas others have reported a moderately strong association^10^. It also accords with existing evidence reporting a negative association between extraversion and physiological responsivity during music listening^75^ and that extraverted people are more likely to use music as a background to other activities, rather than for emotional purposes^76^.

In sum, of the five personality dimensions, we found open-mindedness, agreeableness, and negative emotionality to be most strongly and positively related to musical sensibility. These traits all comprise affective components, including a propensity to experience emotional responses to aesthetic stimuli (open-mindedness), a tendency for negative emotions (negative emotionality), and emotional tendencies toward others (agreeableness)^77^. Correspondingly, musical sensibility, as measured here, also covers a wide spectrum of emotional and aesthetic responses to music, ranging from basic emotional receptivity to more complex, contemplative aesthetic experiences (e.g., feelings of wonder or beauty). Hence, the emerging picture is that personality-related emotional- and aesthetic orientations towards art, others, and self are tied to musical sensibility through a shared affective component. This is further corroborated by the overall weak associations between musical sensibility and conscientiousness, which is the only Big Five trait that is not related to emotional tendencies^77^. Such an ‘affective core’ would support and echo our previous findings and preliminary interpretation of a general musical sensibility factor, wherein we posited that its psychological content could be related to strong emotional and aesthetic responses to music or the degree to which one is able to feel the music^7^.

As suggested by past research, however, the affective bond between personality and musical sensibility is likely to be trait-congruent. That is, personality-related affective styles can influence both the quality and intensity of the music-induced emotional experiences, possibly mediated by different emotion-induction mechanisms. Moving forward, there is a need to get a more nuanced understanding of these emotion-induction processes, or the ‘how*’* of music-elicited emotions^78^ (examples of different theoretical perspectives can be found in e.g.^79–84^), and the potential moderating role of individual factors like personality^78^.

### The role of genetic and environmental factors in the musical sensibility and personality links

As previously shown in the present sample, heritability for general musical sensibility was moderately high (64%), with additional contributions from non-shared environmental influences (36%). Here, results further showed moderate to moderately high heritability estimates for the broad personality domains (47–63%). At the facet level, heritability estimates for compassion (35%) and aesthetic sensitivity (58%) were slightly lower than for the corresponding domains, most likely representing greater measurement error, and thus, an increase in the proportion of non-shared environmental variance^85^.

Most of these heritability estimates are consistent with existing meta-analytic results of personality traits (heritability range 40–50%)^53–55^. For open-mindedness, however, the present estimate was higher (i.e., 63%) than what has been typically reported. That said, a recent cross-national twin study examining age trends in the relative contribution of genetic and environmental factors reported comparable heritability estimates for open-mindedness, ranging from 59% in adolescence to 69% in old age^86^. Also consistent with most existing twin literature in adults^87^, we found that non-shared environmental factors also play an important role, accounting for the remaining variance in each trait, whereas dominant genetic effects and shared environmental influences were non-significant.

Regarding the causes of covariation between traits, our results suggest that genes, relative to non-shared environmental factors, play a key role in all musical sensibility and personality associations, accounting for half or more of the observed covariance between traits. Genetic factors were particularly important for the musical sensibility associations with agreeableness and open-mindedness (100% and 77%, respectively), the latter mirroring the results reported by Butković et al*.*^59^ We also found evidence for substantial genetic overlap between musical sensibility and particularly open-mindedness (*r*A ~ 0.66), whereas the genetic correlations with agreeableness (*r*A ~ 0.26) and negative emotionality (*r*A = 0.13) were comparatively lower. This suggests that the biological mechanisms underlying musical sensibility and open-mindedness are highly overlapping and more similar than those underlying musical sensibility and both agreeableness and negative emotionality. Conversely, the genetic factors underlying the links between musical sensibility and both agreeableness and negative emotionality were largely unique to each trait. Importantly, however, the genetic paths from both dimensions remained significant after accounting for the large effects of open-mindedness.

Overall, the amount of shared genetic and environmental variance with personality-related factors was 28% and 5%, respectively, meaning that of the total variance in musical sensibility, about 33% overlapped with personality. This finding can be interpreted as follows: First, given that these associations were largely genetically driven, our results suggest that parts of the genetic variance underlying individual differences in musical sensibility are also responsible for individual differences in open-mindedness, and, to a lesser extent, agreeableness, and negative emotionality, and/or vice versa. Thus, these findings imply that personality does not cause, nor is it a consequence of musical sensibility, but rather that the traits may have partly overlapping biological underpinnings, possibly through common affective pathways. Nevertheless, we did find significant non-shared environmental influences between musical sensibility and open-mindedness, as well as negative emotionality, suggesting additional contributions of environmental factors to these relationships. It is important to note, however, that our cross-sectional data does not permit any inferences regarding the causality and directionality of these associations.

Secondly, while this percentage seems substantial, there is clearly more to musical sensibility than what can be explained by personality-related factors. Indeed, even though the genetic overlap between musical sensibility and personality, specifically open-mindedness, was relatively high, the phenotypic associations were generally small or moderate. Future research should aim to identify other plausible psychological determinants of musical sensibility, e.g., social-relational traits like empathy, as well as cognitive abilities, attention, and absorption.

Notable strengths of the present study include the use of comprehensive, validated, and reliable instruments in a large population-based twin sample. The study is, of course, also subject to certain limitations. First, our sample consisted largely of middle-aged women from a Scandinavian country. While our sample is likely to be reasonably representative of corresponding populations in other Western countries, the results from this study cannot necessarily be assumed to generalize to all other age groups, cultures, and ethnicities. Second, both personality and musical sensibility were measured using self-reports. Although self-reports represent the most common and important sources of information for these traits, they are also subject to important limitations (e.g., response bias) and may not fully capture e.g., the affective granularity of musical experiences. Future work incorporating alternative behavior-based measures will be useful for providing a comprehensive profile of musical sensibility, as well as the relationship to personality. For example, a recent study^88^ found that physiological synchrony within concert audiences was associated with, e.g., participants’ aesthetic- and affective experiences, modes of listening (e.g., emotional listening), and personality traits. Third, to focus on substantive effects, we only included personality traits and facets with the strongest unique effects in the multivariate twin models, which might have resulted in an incomplete picture. Finally, our study might not have been sufficiently powered to detect small dominance genetic- and shared environmental effects.

## Conclusions

The present study has shown that certain personality traits and facets appear to be important correlates of musical sensibility and may constitute an inherent part of the musical sensibility construct. Indeed, the use of a genetically sensitive design allowed us to gain novel insight into the underlying etiological architecture, demonstrating that genetic factors seem to be the main driver behind these personality-musical sensibility links and, further, that their genetic underpinnings, to varying degrees, overlap. Taken together, the findings make a persuasive case for ascribing affectivity and general aesthetic sensitivity as key characteristics of musical sensibility. As such, our findings help building the growing body of research on individual differences in the affective domain of music. Still, only about one-third of the variance in musical sensibility could be explained by personality-related factors. Given that musical sensibility is likely to play an important role in the way people engage with and experience music, we hope the present findings will stimulate new research into the nature of individual differences in musical sensibility.

## Methods

### Sample

Twins were recruited from the Norwegian Twin Registry (NTR)^89^, a consent-based health registry that serves as a research resource by integrating and maintaining nationwide twin data. The NTR comprises several twin cohorts (an overview can be found in^89^), whereby the cohorts in focus for the present study were same-sex twins born 1967–1991. In total, 7871 twins were invited, of which 2611 individuals signed the informed consent and agreed to participate in the study. The final sample with valid personality- and musical sensibility scores (N = 2592) consisted of 1232 paired responders and 1360 single responders, of which 1719 were women (mean (SD) age = 43.4 (7.75), range = 31–55) and 873 were men (mean (SD) age = 44.4 (7.29), range = 31–55). Of the full twin pairs, there were 119 MZ male pairs, 307 MZ female pairs, 44 DZ male pairs, and 146 DZ female pairs (totaling 616 complete twin pairs).

### Measures

*Personality* was assessed with the BFI-2^27,90^. This 60-item inventory covers the five broad domains, namely, Extraversion, Agreeableness, Conscientiousness, Negative Emotionality, and Open-mindedness, in addition to three facets for each domain (Table 1, block II). Items are rated on a 5-point scale ranging from *strongly disagree* to *strongly agree.* In the current sample, reliability calculated using Cronbach’s α were 0.85 (extraversion), 0.77 (agreeableness), 0.85 (conscientiousness), 0.89 (negative emotionality), and 0.83 (open-mindedness). For the facets, Cronbach’s α ranged from 0.55 (A2 respectfulness) to 0.82 (E1 sociability/N2 depression), with mean α = 0.72. A full overview of all items can be found at: https://www.colby.edu/wp-content/uploads/2013/08/bfi2-item-list.pdf

*Musical sensibility* was measured with module 2 of the MUSEBAQ^15^. It consists of 24 items that measure sensitivity and responsiveness to music across four subscales, that is, emotional sensitivity to music, personal commitment, music memory and imagery, and listening sophistication. Responses are given on a 5-point scale from *strongly disagree* to *strongly agree.* An example item for this scale is “*Tears come to my eyes when listening to some pieces of music*”. Supplementary Table S3 provides an overview of all items. The subscales had good psychometric properties in the present sample (Cronbach’s α range = 0.81–0.91, mean = 0.85). Based on our previous findings^7^, we calculated a global mean score over the total number of items that were used in all analyses (Cronbach’s α = 0.95).

### Statistical analyses

All analyses were performed using *R*^91^ and *R Studio*^92^. Descriptive statistics, correlations, and regression analyses were estimated based on data from single twins and one member from each twin pair (*N* with valid personality and musical sensibility scores = 1979) to minimize bias.

#### Correlation- and regression analyses

First, bivariate correlations were used to assess the relationship among the personality domains, their corresponding facets, and the global musical sensibility score. Next, we used two sets of regression analyses to (1) assess the unique contributions of each of the five personality domains to the musical sensibility-personality relationship, and (2) identify the facets that are most significant for this relationship. Age and sex were included as covariates in both regression models.

#### Multivariate twin analyses

The CTD, which includes MZ and DZ twins reared together, was used to estimate the genetic and environmental influences on the variation in and covariation between measures. Whereas MZ twins share all of their genetic material, DZ twins share on average 50% of their genetic makeup^93^. In multivariate applications of the CTD, information on this genetic relatedness allows for the decomposition of variance and covariance into additive (A) genetic factors, non-additive (D; dominance) genetic factors, shared environmental influences (C; i.e., influences that are common or shared between both members of a twin pair and contribute to within-pair similarities), and non-shared environmental effects (E; i.e., individual-specific effects that do not contribute to within-pair similarities, in addition to measurement error). The D and C variance components are confounded in the CTD and can therefore not be estimated simultaneously. As a heuristic, if the MZ within-pair (intraclass) correlations are less than twice the DZ intraclass correlation, shared-environmental influences are inferred, whereas dominance genetic effects are indicated when the MZ intraclass correlations are more than twice the DZ intraclass correlations.

Genetic model fitting was conducted using *umx*^94^ and *OpenMx*^95^ with full information maximum likelihood estimation. Prior to genetic modeling, assumptions of the classical twin design were tested by constraining means and variances to be equal within pairs and across zygosity. Since an investigation of potential sex differences in the etiological architecture was beyond the scope of this study, scores were residualized for the effects of sex and age before fitting genetic models to avoid potential parameter bias^61^.

To elucidate the genetic and environmental contributions to the associations between personality and musical sensibility, two sets of multivariate Cholesky models^96^ were performed, assessing: (1) the relationship between the Big Five *domains* and musical sensibility, and; (2) the relationship between the specific Big Five *facets* and musical sensibility. We first fitted a full (saturated) ACE and ADE Cholesky model, which was then compared to progressively more restricted models (i.e., an AE and E model). Goodness-of-fit and model comparison was evaluated by a minus2LogLikelihood difference test (Δ − 2LL) and AIC^97^, whereby lower AIC values indicate better fit. The more parsimonious model is preferred when the nested model does not cause a significant reduction in goodness-of-fit, as indexed by a non-significant *p-value,* relative to the saturated model^98^.

## Supplementary Information

Supplementary Information.

## Data Availability

The dataset used here is not publicly available but may be requested from the Norwegian Twin Registry. Restrictions apply, however, as the dataset was used under a license obtained for the present study. More information about data access can be found here: https://www.fhi.no/hs/tvilling/tvillingregisteet/
