# Supplementary material for: The role of Big Five personality domains and facets in musical sensibility: a twin study
Source: Sci Rep. 2025 Apr 19;15:13559. doi: 10.1038/s41598-025-95661-z (PMC12009312; doi:10.1038/s41598-025-95661-z)
Supplement: Supplementary file 1 — Supplementary Information. [file 41598_2025_95661_MOESM1_ESM.docx]

**SUPPORTING MATERIALS.**

**Table S1**: Tests of the assumptions of equal means and variances across twin order and zygosity for the two multivariate biometric models. Models 2-4 are compared with the fully saturated model 1, showing no significant effects of constraining means and variances (all *p’s* > .150).

**
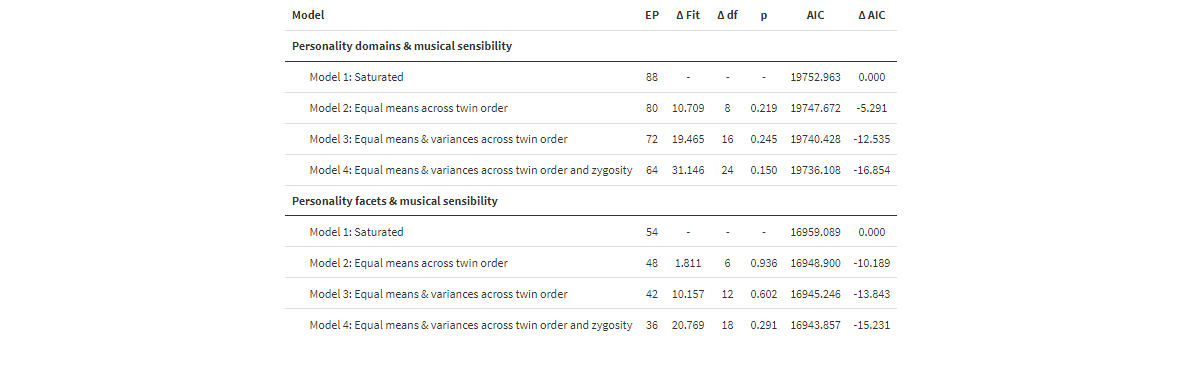
**Abbreviations: *AIC* Akaike Information Criterion; *df* Degrees of Freedom; *EP* Estimated Parameters; *Fit* − 2LL negative two times the log-likelihood.

**Table S2:** Standardized genetic (A) and unique environmental (E) paths and confidence intervals in brackets derived from the best-fitting AE model comprising all personality domains and the global musical sensibility score.

**Table S3**: Overview of descriptive statistics for items and subscales of Module 2 of the MUSEBAQ, derived from a sample consisting of single twins and one individual from each full twin pair.

| Subscales and items | Item | *M* | *SD* | Skewness | Kurtosis | α |
| --- | --- | --- | --- | --- | --- | --- |
| *Emotional Sensitivity* |  | 3.59 | 0.81 | -0.62 | 0.11 | 0.91 |
| I experience strong emotions when I listen to particular types of music | 2 | 3.92 | 1.03 | -0.89 | 0.34 |  |
| I tend to appreciate music for its beauty or sublimity | 6 | 3.88 | 0.94 | -1 | 1.11 |  |
| I get chills or gooseflesh when listening to moving music | 9 | 3.92 | 1.08 | -1.04 | 0.53 |  |
| Tears come to my eyes when listening to some pieces of music | 10 | 3.47 | 1.31 | -0.51 | -0.89 |  |
| Music can produce feelings of wonder and fascination in me | 13 | 3.34 | 1.15 | -0.44 | -0.59 |  |
| I can’t help swaying my body or tapping my foot when listening to some music | 15 | 3.77 | 1.13 | -0.85 | -0.04 |  |
| When I listen to live music, I tend to experience the emotions expressed by the performers. | 18 | 3.38 | 1.06 | -0.57 | -0.20 |  |
| I sometimes seem to ‘catch’ the emotions that other listeners experience while listening to music | 19 | 2.97 | 1.06 | -0.22 | -0.69 |  |
| I can be greatly moved by music | 23 | 3.48 | 1.16 | -0.6 | -0.42 |  |
| Listening to music fills me with emotion | 24 | 3.75 | 0.95 | -0.79 | 0.60 |  |
|  |  |  |  |  |  |  |
| *Personal Commitement* |  | 2.64 | 0.86 | 0.15 | -0.50 | 0.85 |
| I often spend time online or in shops looking for music | 4 | 2.42 | 1.21 | 0.46 | -0.80 |  |
| I couldn’t live without music | 8 | 3.52 | 1.22 | -0.5 | -0.71 |  |
| It’s important for me to choose each piece of music I listen to | 12 | 2.47 | 1.09 | 0.37 | -0.54 |  |
| It’s important that I give my full attention to music when listening | 16 | 2.58 | 1.07 | 0.35 | -0.50 |  |
| Music is like an addiction for me | 21 | 2.72 | 1.17 | 0.18 | -0.81 |  |
| I become so involved in music I’m listening to that I lose track of time or where I am | 22 | 2.14 | 1.02 | 0.72 | -0.06 |  |
|  |  |  |  |  |  |  |
| *Music Memory & Imagery* |  | 3.35 | 0.97 | -0.37 | -0.43 | 0.83 |
| I find it difficult to stop reliving my past when I listen to some music | 3 | 3.55 | 1.2 | -0.65 | -0.48 |  |
| I often see detailed pictures or movies in my head when I listen to some music | 7 | 3.01 | 1.26 | -0.1 | -1.04 |  |
| Music often evokes vivid memories from my past | 14 | 3.62 | 1.11 | -0.75 | -0.06 |  |
| Images appear without any effort when I hear some music | 17 | 3.2 | 1.17 | -0.29 | -0.83 |  |
|  |  |  |  |  |  |  |
| *Listening Sophistication* |  | 3.04 | 0.93 | -0.18 | -0.58 | 0.81 |
| After hearing a new song a few times, I can usually sing or hum it by myself. | 1 | 3.43 | 1.23 | -0.49 | -0.76 |  |
| I am able to describe a piece of music I’ve heard to someone else | 5 | 2.51 | 1.16 | 0.27 | -0.87 |  |
| I’m intrigued by music I’m not familiar with and want to find out more | 11 | 2.97 | 1.13 | -0.1 | -0.74 |  |
| I have a good ear for music | 20 | 3.26 | 1.14 | -0.34 | -0.59 |  |
|  |  |  |  |  |  |  |
| *Musical sensibility (global score)* |  | 3.15 | 0.77 | -0.27 | -0.37 | 0.95 |

Abbreviations: *M* Mean; *SD* Standard deviation; *α* Cronbach’s alpha.
